# Phase-PCANet for Fingerprint Image Liveness Detection

**DOI:** 10.3390/jimaging12090422

**Published:** 2026-09-08

**Authors:** Jing Li, Xinqi Wang

**Affiliations:** Information Science Department, Xi’an University of Technology, Xi’an 710048, China

**Keywords:** liveness detection, local phase information, global phase information, PCANet

## Abstract

This paper proposes Phase-PCANet, a new image representation method for fingerprint liveness detection. Phase-PCANet integrates both local and global phase features. The local phase, capturing fine-grained edges and textures, is extracted via short-time Fourier transform with singular value decomposition; the global phase, encoding the holistic structural layout, is obtained from a full-image Fourier transform. These two phase components are separately fed into an improved PCANet, which employs dual binary coding, i.e., scalar-based intra-channel coding and vector-similarity-based inter-channel coding, to preserve within-channel structures and cross-channel correlations. Multi-stage features from different PCA layers within each phase path are aggregated, and the resulting deep features from both paths are concatenated to form the final image representation. Experiments on the LivDet 2011, 2013, and 2015 databases verify the effectiveness of the proposed method.

## 1. Introduction

Being a fundamental problem in pattern recognition and computer vision, image liveness detection plays a critical role in many applications. Feature extraction lies at the core of this process, as effective feature extraction not only reduces the computational complexity of classification algorithms but also improves recognition performance.

Image complex-valued transformation is an efficient approach for image feature extraction. The magnitude and phase components are two distinct representations derived from this transformation, with the phase component encoding critical structural and perceptual information that is often more significant than the magnitude. Oppenheim et al. [1] demonstrated that, in signal reconstruction, the phase information of a signal is more informative than its magnitude.

Inspired by principal component analysis (PCA), PCANet is an unsupervised learning network with a straightforward and rule-based architecture. Compared with end-to-end deep convolutional neural networks (CNNs), PCANet offers several distinct advantages in specific scenarios. First, its architecture is entirely data-driven and closed-form, requiring no backpropagation or iterative gradient-based optimization, which eliminates the need for careful hyperparameter tuning (e.g., learning rates, weight initialization, and dropout) and ensures a deterministic and reproducible feature extraction process. Second, PCANet has substantially fewer trainable parameters, making it computationally efficient and amenable to small-sample learning, where deep CNNs typically suffer from severe overfitting. Third, the cascade of PCA-based filter banks provides an interpretable linear subspace representation, facilitating theoretical analysis of the learned features and their relationships to input data statistics—a property often lacking in black-box deep models. Finally, PCANet’s simple multilayer architecture can be implemented rapidly with basic linear algebra operations, enabling fast prototyping and deployment on resource-constrained platforms without sacrificing competitive performance.

Despite the progress made by existing methods, several limitations remain unaddressed. First, while phase information has been recognized as a powerful representation for image analysis, most existing approaches exploit either the local phase or global phase in isolation. The local phase captures fine-grained edge and texture details but lacks holistic structural context, whereas the global phase encodes the overall layout and periodicity of fingerprint patterns but may overlook subtle material-induced micro-anomalies. The complementary nature of these two phase representations has not been fully exploited for fingerprint liveness detection. Second, conventional PCANet employs a simple threshold-based hashing scheme that converts filter responses into binary codes without preserving the spatial structure within each channel or the correlations across channels. This limits the discriminative power of the learned features, particularly in distinguishing subtle textural differences between live and spoof fingerprints. Third, most existing handcrafted feature descriptors rely on a single encoding strategy and a single feature fusion level, which may fail to capture the multi-scale and multi-level characteristics inherent in fingerprint images. These limitations motivate us to explore a unified framework that jointly leverages local and global phase information within an improved PCANet architecture.

To this end, we propose Phase-PCANet, a new feature extraction method for fingerprint image liveness detection. The main contributions of this work are threefold:

1. Joint exploitation of local and global phase information. Unlike existing methods that rely on either the local or global phase alone, Phase-PCANet extracts the local phase via STFT combined with SVD to capture fine-grained edge and texture details, and the global phase via full-image DFT to encode the holistic structural layout. This dual-path design enriches the discriminative information available for liveness detection.
2. Improved PCANet with dual binary coding on each phase path. The local and global phase components are separately fed into an improved PCANet. Within each path, we design two complementary binary coding strategies: (i) an intra-channel contrast binary pattern (CBP_1_) that captures structural variations within each filter response channel using a scalar ratio threshold, and (ii) an inter-channel binary pattern (CBP_2_) that encodes cross-channel correlations via angular similarity between feature vectors. This overcomes the limitation of conventional PCANet hashing, which ignores both intra-channel spatial structure and inter-channel relationships.
3. Multi-level feature aggregation. Feature fusion is performed at two complementary levels. At the intra-path level, multi-stage features from different PCA layers of the improved PCANet are aggregated within each phase path, enriching the representation with both low-level detail and high-level semantic information. At the inter-path level, the deep features independently learned from the local and global phase paths are concatenated to form the final image representation.

The rest of this paper is organized as follows. In Section 2, we briefly review the work on fingerprint liveness detection. In Section 3, we describe our proposed Phase-PCANet image representation method in detail. Section 4 presents the experimental results on three databases. Finally, we conclude the paper in Section 5.

## 2. Related Work

Fingerprint liveness detection serves to separate live fingerprint images from their counterfeit counterparts across varying presentation conditions. In particular, the detection techniques are broadly grouped into hardware-based and software-based solutions.

Hardware-based fingerprint liveness detection relies on specialized sensors—such as optical coherence tomography (OCT) or multi-spectral imaging devices—to capture intrinsic physiological signs of living tissue, including blood flow, pulse oximetry, temperature, and even odor. While such approaches can achieve high detection accuracy by directly probing the physical properties of the finger, they entail significant drawbacks: increased system complexity, higher hardware costs, and limited upgradeability, as any improvement typically requires physical sensor replacement rather than software updates.

In contrast to hardware solutions, software-based fingerprint liveness detection operates solely on images captured by standard sensors, requiring no additional devices. This confers distinct advantages in cost and upgrade flexibility, which have fueled increasing scholarly attention. Within this category, methods are further divided into conventional handcrafted feature-based techniques and deep learning-based approaches.

### 2.1. Traditional Feature Analysis Methods

Traditional feature analysis liveness detection methods differentiate live from spoof fingerprints by examining intrinsic features in the captured images, such as sweat pores, skin elasticity, perspiration patterns, and overall image quality.

Several studies have pursued pore-based liveness detection from different angles. Manivanan et al. [2] pioneered the use of high-pass and correlation filtering to extract and localize active sweat pores. The discriminative value of pore quantity was subsequently corroborated by Espinoza and Champod [3], who reported significant differences between genuine and spoof samples. In a more recent contribution, Lu et al. [4] combined wavelet transform with Gaussian filtering for pore extraction, characterized pore distribution through statistical descriptors, and employed SVM for final classification. A common methodological bottleneck across these approaches is the stringent requirement for high image resolution to ensure reliable detection of minute pore features.

Antonelli et al. [5] devised an elasticity-based approach to examine the mechanical response of live skin tissue for liveness detection. Zhang et al. [6], in parallel, formulated a thin-plate spline model driven by bending energy vectors to discriminate between authentic and fake fingerprints. Jia et al. [7] adopted a different strategy, utilizing the correlation coefficient and standard deviation of image-sequence features as discriminative cues for spoof detection.

Abhyankar and Schuckers [8] proposed a wavelet-based approach to detect perspiration variations along fingerprint ridges for liveness detection. Despite its conceptual merit, this method suffers from several practical limitations: the perspiration features are difficult to capture reliably, require active user cooperation, are inherently time-consuming, and are thus ill-suited for real-time deployment. Abhyankar and Schuckers [9] proposed a fast, single-image liveness detection algorithm that combines multiresolution texture features and cross-ridge frequency statistics with a fuzzy c-means classifier for efficient live-versus-fake fingerprint discrimination.

Considering that the surface of a live finger is generally smoother than that of a spoof model, and that spoof fingerprints tend to produce lower-quality images, image-quality-based methods have been adopted for liveness detection. Galbally et al. [10] proposed a liveness detection approach leveraging ten quality-related features. Park et al. [11] introduced a representation method based on six statistical moments of fingerprint images. Similarly, Sharma and Dey [12] extracted eight quality features from ridge-valley structures of fingerprint images. Yuan et al. [13] employed horizontal and vertical difference co-occurrence matrices to distinguish between genuine and fake fingerprints. Moon et al. [14] applied wavelet analysis to the texture of the fingertip surface for fingerprint liveness detection. Pereira et al. [15] improved the wavelet analysis of the fingertip surface texture by introducing spatial features to the spatial surface coarseness analysis.

Image classification-based liveness detection methods frame the detection problem as a binary classification task between live facial images and spoof facial images, which primarily involves two key aspects: image feature extraction and classification. Nikam and Agarwal [16] applied ridgelet energy and co-occurrence signatures to represent fingerprint texture features. Nikam and Agarwal [17] used curvelet transform energy and co-occurrence signatures. Ghiani et al. [18] applied the local phase quantization (LPQ) descriptor for the fingerprint image representation. Jia et al. [19] proposed two multiscale local binary pattern (LBP) descriptors, by increasing the radius of the operator or applying a set of filters, to extract wider spatial support area image texture features. Gottschlich et al. [20] proposed histograms of invariant gradients (HIG) as a ridge texture descriptor for the fingerprint image. Its gradient-position-independent estimation offers a promising solution to cross-device liveness detection. Zhang et al. [21] applied wavelet analysis to generate denoised and residual noise images and extracted LBP histograms from their blocks as enriched texture features of the fingerprint image. Gragnaniello et al. [22] applied a wavelet-Markov local descriptor exploiting joint dependencies among wavelet coefficients for fingerprint image representation. Rattani and Ross [23] combined linear and non-linear image denoising methods to reduce the impact of fake fingerprint fabrication material type. Rattani and Ross [24] addressed the issue of novel spoof materials by fusing a novel-material detector with a fingerprint liveness detector. Gragnaniello et al. [25] analyzed the images in both the spatial and frequency domains by applying the Weber local descriptor (WLD) and LPQ, respectively. The subsequent fusion of these two descriptors further improved the detection accuracy. Dubey et al. [26] proposed score-level fusion of the speeded-up robust features (SURF), pyramid histograms of oriented gradients (PHOG) and Gabor transform texture features for fingerprint image representation. For image representation, Xia et al. [27] quantized each image, computed pixel-wise gradients with magnitude truncation, and constructed second- and third-order co-occurrence matrices from the truncated gradients. Xia et al. [28] proposed a feature extraction method that fuses spatial-domain WLBP descriptors with frequency-domain circularly symmetric Gabor features. Motivated by the observation that gradient-field directional coherence effectively discriminates live from fake fingerprints, Kim [29] proposed the local coherence pattern (LCP) descriptor, which captures this coherence to represent the intrinsic ridge-valley structure. Ghiani et al. [30] adopted binarised statistical image features (BSIFs) to encode fingerprint image. Yuan et al. [13] proposed a fingerprint representation method that constructs gradient co-occurrence matrices along the horizontal and vertical directions, respectively. Mehboob et al. [31] proposed a rotation-invariant fingerprint image representation method that independently quantizes and fuses global Shepard magnitude features (from spatial-domain edge variations) and local phase-based features (from dominant frequency components). Park et al. [11] combined PDF-derived statistical moments (mean, variance, skewness, kurtosis, hyperskewness, and hyperflatness) with average brightness, standard deviation, and difference for fingerprint image representation. Alshdadi et al. [32] proposed a fundamental fingerprint descriptor that constructs a 2-D histogram from quantized ridge contours and ridge orientations. Gonzalez-Soler et al. [33] extracted dense-SIFT descriptors via PHOW, encoded them in parallel using BoW, FV, and VLAD, and concatenated the three feature vectors for image representation. Sharma and Selwal [34] proposed a descriptor, termed Local Adaptive Binary Pattern (LABP), which computes binary codes using a dynamically adapting threshold. The LABP features are then concatenated with existing CLBP and BSIF descriptors for fingerprint image representation. Sharma and Selwal [35] proposed a fingerprint image representation method that combines LABP with uniform local binary patterns (ULBP). Agarwal and Bansal [36] extracted and fused pore-based and texture-based features for fingerprint image representation. Mehboob and Dawood [37] proposed DEHFF, a fingerprint descriptor that extracts ridge contour information via spatial stimulus responses and Gabor transforms, and estimates ridge orientation using derivative filters and LPQ. Yuan et al. [38] applied the LBP coding method to enhance the ridge features of the fingerprint image.

Commonly used classifiers for fingerprint liveness detection include Support Vector Machine (SVM) [11,21,27,29,31,33], AdaBoost [35], and Backpropagation (BP) neural networks [13].

### 2.2. Deep Learning Methods

Deep learning methods have achieved state-of-art performance in fingerprint liveness detection in recent years. Menotti et al. [39] applied a convolutional neural network (CNN) with architecture and filter optimization for fingerprint liveness detection. Kim et al. [40] developed a fingerprint liveness detection approach using a deep belief network (DBN). The method first locates the regions of interest (ROI) in fingerprint images, from which small patches are extracted, normalized, and vectorized. These vectors are subsequently transformed by the DBN into high-dimensional feature representations for liveness classification. Zhang et al. [41] proposed a hybrid approach combining fingerprint matching and liveness detection. Their method computes a matching score between two images and a liveness score via a modified Slim-ResCNN. The two scores are fused and then classified by logistic regression to differentiate live from fake fingerprints. Yuan et al. [42] proposed a multimodal feature fusion strategy that integrates complementary features from multiple distinct CNN backbones for fingerprint liveness detection. Kothadiya et al. [43] developed an attention-enhanced ResNet for fingerprint liveness detection, incorporating both channel and spatial attention mechanisms. Zhang et al. [44] proposed a ResNet and Transformer-based fingerprint liveness detection method, which performs foreground extraction, divides the image into local patches, and finally utilizes a hybrid architecture to make the final liveness decision.

## 3. Phase-PCANet Image Feature Learning

The proposed method leverages the local phase feature, global phase feature, and an improved PCANet for deep feature learning. It effectively captures informative content embedded in fingerprint images. The methodology is detailed as follows.

### 3.1. Image Local Phase Feature Extraction

The local phase feature is extracted using the short-time Fourier transform (STFT) and singular value decomposition (SVD), as described below. Firstly, apply the STFT over a square neighbourhood at each pixel position *n* of the image I(n) as follows:

(1) $$F\left(u\right)=\frac{1}{N}\sum_{n=0}^{N-1}I\left(n\right)e^{-i2\pi un/N},$$

*i* is the imaginary unit. I(n) is a vector containing all the *N* image pixel samples from the neighborhood. A set of multiple coefficients *u* (e.g., u=0,1,⋯,N−1), each corresponding to a 1D frequency, is selected for STFT computation in (1) to obtain a comprehensive representation of the local image.

Secondly, the STFT matrix of the center pixel is defined as

(2) G=⋮⋮Re(F(u))Im(F(u))⋮⋮,

where Re(F(u)) and Im(F(u)) denote the real and the imaginary parts of a complex number F(u), respectively.

Thirdly, the covariance matrix of the STFT matrix of multiple frequency responses is defined as

(3) C=GTG=∑Re(F(u))Re(F(u))∑Re(F(u))Im(F(u))∑Im(F(u))Re(F(u))∑Im(F(u))Im(F(u)).

Finally, perform SVD of *G*:

(4) $$G=USV^{T}=U\mathrm{diag}\left[s_{1},s_{2}\right]{\left[v_{1},v_{2}\right]}^{T},$$

where *U* is a P×2 matrix. *S* is a diagonal 2×2 singular value matrix representing the energy. *V* is a 2×2 matrix, and its first column vector $v_{1}=\left[v_{1,1},v_{1,2}\right]$ describes the dominant phase of the local STFT field. The local phase feature is defined as:

(5) $$\mathrm{LP}\left(n\right)=\arctan2\left(v_{1,1},v_{1,2}\right).$$

### 3.2. Image Global Fourier Phase Feature Extraction

For an image $I\in R^{M\times N}$, where *M* and *N* represent the height and width, respectively, the 2D Discrete Fourier Transform (DFT) is applied to transform the image into the frequency domain:

(6) $$F\left(u,v\right)=\sum_{m=1}^{M}\sum_{n=1}^{N}I\left(m,n\right)\exp\left(-i2\pi \left(\frac{m}{M}u+\frac{n}{N}v\right)\right),$$

where $\left(m,n\right)$ and $\left(u,v\right)$ represent coordinates in the spatial and frequency domains, respectively. *i* is the imaginary unit. The complex-valued function F(u,v) can be expressed as:

(7) F(u,v)=ReF(u,v)+iImF(u,v),

Re$\left(F(u,v)\right)$ and Im$\left(F(u,v)\right)$ are the real and imaginary components of the Fourier function F(u,v), respectively. The global phase feature GP(u,v) is defined as

(8) GP(u,v)=arctan2ImF(u,v),ReF(u,v).

### 3.3. Improved PCANet Feature Learning

**Figure 1:**
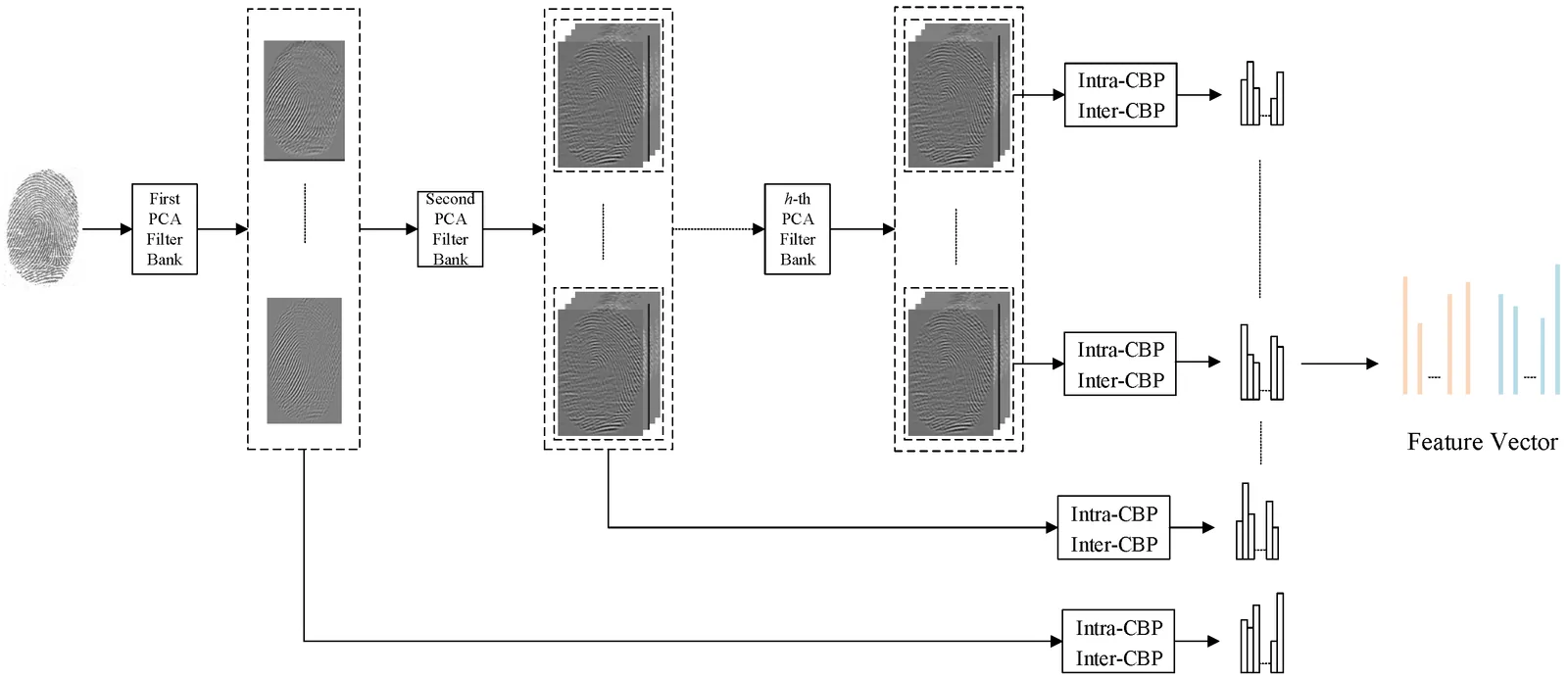
Principle structure diagram of the improved PCANet.

The improved PCANet consists of three main components: cascaded principal component analysis (PCA), binary coding, and block-wise histograms. Figure 1 depicts the structure of the improved PCANet.

**First Step: PCA Filtering**

Assume that there are *N* learning samples of size m×n. For a given sample $X_{j}$, a patch of size $k_{1}\times k_{2}$ is taken around each point. By subtracting the patch mean from each patch, a new matrix is arranged as ${\hat{X}}_{j}=\left[{\hat{X}}_{j1},{\hat{X}}_{j2},\ldots ,{\hat{X}}_{j\left(m-k_{1}+1\right)\times \left(n-k_{2}+1\right)}\right]$, where ${\hat{X}}_{jk}\in R^{k_{1}\times k_{2}}$ denotes the *k*-th mean-removed patch in ${\hat{X}}_{j}$. Then, all training samples are vectorized and integrated in the following manner:

(9) $$\hat{X}=\left[{\hat{X}}_{1},{\hat{X}}_{2},\ldots ,{\hat{X}}_{N}\right].$$

Subsequently, the eigenvectors of $\hat{X}{\hat{X}}^{T}$ are computed and $L_{1}$ principal eigenvectors are selected as PCA filters $W_{l}$:

(10) $$W_{l}={\mathrm{MAT}}_{k_{1},k_{2}}\left(Q_{l}\left(\hat{X}{\hat{X}}^{T}\right)\right)\in R^{k_{1}\times k_{2}},\;l=1,2,\ldots ,L_{1},$$

where $Q_{l}\left(\hat{X}{\hat{X}}^{T}\right)$ denotes the *l*-th principal eigenvector of $\hat{X}{\hat{X}}^{T}$, and MAT(v) is a function that maps the vector $v\in R^{k_{1}k_{2}}$ to a matrix $W\in R^{k_{1}\times k_{2}}$.

The output of the *l*-th filter at the first PCA stage is expressed as:

(11) $$Y_{i}^{l}=X_{i}*W_{l},\;i=1,\ldots ,N,$$

where ∗ represents the 2D convolution operator.

The second and subsequent PCA stages follow the same pipeline as the first, with each stage taking the filter response maps from its predecessor as input. For the second stage, the output is obtained as:

(12) $$Z_{i}^{l^{\prime }}=Y_{i}^{l}*W_{l^{\prime }}^{2},\;i=1,\ldots ,N,l^{\prime }=1,2,\ldots ,L_{2},$$

where $L_{2}$ is the number of filters in the second PCA stage. The same procedure applies recursively to deeper stages, with each stage learning its own PCA filters from the patch distributions of the preceding stage’s outputs.

**Second Step: Binary Coding**

The Contrast Binary Pattern (CBP) is proposed to encode the outputs from each PCA stage, enabling the synchronous acquisition of intra-channel and inter-channel features. Intra-channel feature extraction is performed on each channel using a scalar-based contrast binary coding method. For inter-channel features, a feature vector similarity metric is defined to extract the correlational information among different channels.

The Intra-Channel Contrast Binary Pattern (CBP_1_) descriptor is constructed on each PCA-filtered output channel separately. For a given pixel $Z_{c}^{l}$ with *N* neighbors $Z_{n}^{l}$ of channel *l*, n=0,…,N−1, the Intra-CBP value is calculated as

(13) $${\mathrm{CBP}}_{1}\left(Z_{c}^{l}\right)=\sum_{n=0}^{N-1}S\left(\frac{|Z_{n}^{l}-Z_{c}^{l}|}{Z_{c}^{l}}-t\right)\cdot 2^{n},$$

where S(x)=1, if x≥0; else S(x)=0. When $Z_{c}^{l}$ = 0, we set the ratio to 0 to avoid division by zero. The proportional constant *t* is utilized as the threshold to separate the neighbors into two categories, and here *t* is set to 0.15. That is to say, the neighbor $Z_{n}$ is regarded as a high perception point if $\frac{|Z_{n}^{l}-Z_{c}^{l}|}{Z_{c}^{l}}\geq t$. Otherwise, $Z_{n}^{l}$ is considered a low perception point.

The Inter-Channel Contrast Binary Pattern (CBP_2_) is computed by comparing the filtered response at a given location with the average filtered response in its surrounding neighborhood. The PCA-filtered response is represented as vector $Z=(z^{1},z^{2},\cdots ,z^{L})$, consisting of *L* of values corresponding to the *L* eigenvector. Specifically, we first compute the angular difference θ between the two response vectors:

(14) $$\theta =\arccos\left(\frac{\langle Z,\bar{Z}\rangle }{∥Z∥\cdot ∥\bar{Z}∥}\right),$$

where Z is the response of the current pixel, $\bar{Z}$ is the average response of the neighborhood, and 〈·,·〉 and ∥·∥ denote the inner product and the Euclidean norm, respectively. When the denominator is null, we simply set θ=0.

Then, θ is linearly quantized into *T* Inter-CBP as follows:

(15) $${\mathrm{CBP}}_{2}\left(Z_{c}\right)=\min\left(\left\lfloor \frac{\theta }{\pi /T}\right\rfloor ,T-1\right).$$

Here, ⌊x⌋ denotes the floor function that returns the largest integer less than or equal to *x*, and the min(·) operator ensures that the maximum angular difference π is mapped to the highest quantization level T−1. Thus, ${\mathrm{CBP}}_{2}\in \left\{0,1,\ldots ,T-1\right\}$, yielding a total of *T* quantization levels. In this work, we set T=8, resulting in 8 quantization levels (0 through 7). An Inter-CBP map is formed by computing the Inter-CBP at all locations in the image.

**Third Step: Image Region CBP Description**

The image CBP representation is the co-occurence of CBP_1_ and CBP_2_ as in Equation (16). For such a consideration, the CBP_1_ and CBP_2_ coding methods are applied to the PCA-filtered output response image set.

(16) $$H\left(r,e\right)=\sum_{m=0}^{M}\sum_{n=0}^{N}\Phi \left({\mathrm{CBP}}_{1}\left(I_{mn}\right)=r\right)\Phi \left({\mathrm{CBP}}_{2}\left(I_{mn}\right)=e\right)$$

Here, M×N is the dimensionality of the image, and $I_{mn}$ is the pixel at location (m,n) in the image coordinates. The number of Intra-CBP and Inter-CBP as mentioned above are *C* and *T*, respectively, and

(17) $$\Phi \left(x\right)=\left\{\begin{array}{cc} 1, & x\;\mathrm{is}\;\mathrm{true},\\ 0, & \mathrm{otherwise}. \end{array}\right.$$

The same CBP encoding and block-wise histogram extraction are applied to all filter response channels from both PCA stages: $L_{1}$ channels from the first stage and $L_{1}\times L_{2}$ channels from the second stage. The histograms from all channels and all blocks are concatenated into a single feature vector, yielding a final dimensionality of $D=\left(L_{1}+L_{1}L_{2}\right)\times R\times \left(C\times T\right)$, where *R* is the number of blocks, *C* is the number of ${\mathrm{CBP}}_{1}$ patterns, and *T* is the number of ${\mathrm{CBP}}_{2}$ quantization levels.

### 3.4. Phase-PCANet Image Representation

Let *I* denote an input fingerprint image. The local phase feature map LP(I) and the global phase feature map GP(I) are first extracted according to Equations (5) and (8), respectively.

Subsequently, both phase representations are independently fed into the improved PCANet. For each phase input P∈{LP(I),GP(I)}, the improved PCANet performs two-stage PCA filtering, intra-channel and inter-channel binary coding, and block-wise histogram aggregation, producing a deep feature vector:

(18) $$\mathrm{PF}\left(P\right)=\Psi_{\mathrm{PCANet}}\left(P\right)\in R^{D},$$

where $\Psi_{\mathrm{PCANet}}\left(\cdot \right)$ denotes the entire improved PCANet feature extraction pipeline, and *D* is the dimensionality of the resulting feature vector.

Finally, the deep feature vectors learned from the local phase and global phase are concatenated to form the overall image representation:

(19) $$\mathrm{PF}\left(I\right)=\left[\;\mathrm{PF}(\mathrm{LP}(I)),\;\mathrm{PF}(\mathrm{GP}(I))\;\right]\in R^{2D}.$$

This concatenated feature vector PF(I) integrates complementary discriminative information: the local phase preserves fine-grained structural details such as edges and ridge textures, while the global phase encodes the holistic geometric layout of the fingerprint.

## 4. Experiments

### 4.1. Databases

Three fingerprint liveness detection benchmark databases, i.e., LivDet 2011, LivDet 2013 and LivDet 2015, are used to evaluate the performance of our proposed method.

In the LivDet 2011 database [45], fingerprints are acquired by four different sensors, i.e., Biometrika, Italdata, Digital Persona, and Sagem. Fake fingerprints are reproduced by the consensual method by using high-quality spoof materials such as ecoflex, wood glue, gelatin, silicon and latex.

In the LivDet 2013 database [46], fingerprints are captured by four different devices, i.e., Biometrika, Crossmatch, Italdata, and Swipe. Fingerprints captured by Biometrika and Italdata are fabricated by a non-cooperative approach using different spoof materials such as gelatin, latex, Play-Doh, wood glue, body double and modasil.

In the LivDet 2015 database [47], fingerprints are captured by four different devices, i.e., Biometrika, Crossmatch, Digital Persona, and Green Bit. The testing set of LivDet 2015 consists of fingerprints reproduced by unknown spoof materials in addition to the known materials (that are also present in the training set). Table 1 presents the characteristic features of three databases.

### 4.2. Evaluation Metrics

After the features are extracted, support vector machine (SVM) is used to distinguish fake fingerprints from real fingerprints. In the training procedure, we choose the Gaussian RBF as the kernel to map the feature vector to a higher-dimensional space to find the optimal line or plane to separate the two classes.

The performance of liveness detection is measured using the average classification error rate (ACE). ACE is the average of false positive rate (FPR) and false negative rate (FNR), as follows:

(20) $$ACE=\frac{FPR+FNR}{2}$$

The equations to calculate FPR and FNR are given below.

(21) $$FPR=\frac{FP}{FP+TN}$$

(22) $$FNR=\frac{FN}{FN+TP}$$

where FP is false positive, TN is true negative, FN is false negative, and TP is true positive. In general, the smaller the ACE is, the better the detection performance of the corresponding method.

### 4.3. Parameter Analysis

There are three important parameters in Phase-PCANet: the number of stages, the number of filters in each layer ($L_{1}$, $L_{2}$, ⋯), and the size of filters ($k_{1}$, $k_{2}$). In this subsection, we focus on the problem of parameter setting for Phase-PCANet on the Biometrika subsection of the LivDet 2015 database.

**Figure 2:**
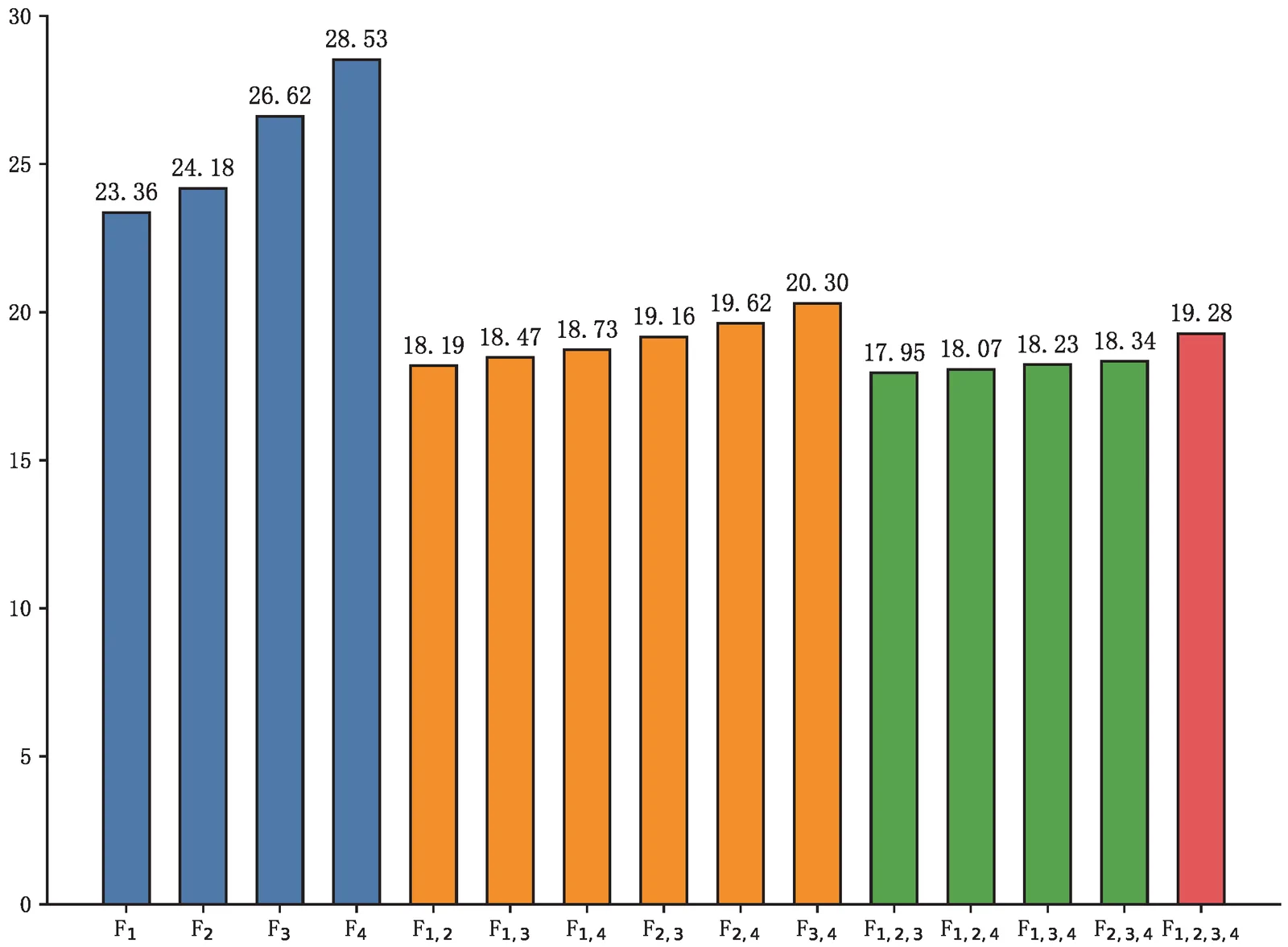
ACE (%) of Phase-PCANet with different stage features.

Initially, we determine the required number of stages in Phase-PCANet and simultaneously evaluate the fusion performance of features from different stages. Accordingly, we vary the number of stages from 1 to 4 and adopt a notation where numerical subscripts represent the PCANet stage indices. For example, $F_{1}$ indicates features extracted from Stage 1 only, and $F_{1,4}$ denotes the concatenated features from Stage 1 and Stage 4. The number of filters in Phase-PCANet stages is fixed at ($L_{1}$,$L_{2}$,$L_{3}$,$L_{4}$)=(5,5,4,4), with fixed filter size 5 × 5. The experimental results are illustrated in Figure 2.

The experimental results reveal three observations. First, shallower stages are consistently more discriminative than deeper ones: single-stage ACE increases from 23.36% ($F_{1}$) to 28.53% ($F_{4}$), and fused features follow the same trend (e.g., $F_{1,2}$: 18.19% vs. $F_{3,4}$: 20.30%). Second, feature fusion enhances performance: multi-stage combinations outperform single stages, with $F_{1,2}$ achieving 18.19% and $F_{1,2,3}$ reaching the best at 17.95%. Third, excessive fusion is detrimental: the full four-stage fusion degrades to 19.28%, likely due to redundancy. Considering computational efficiency, we select the two-stage configuration $F_{1,2}$ for subsequent experiments.

**Figure 3:**
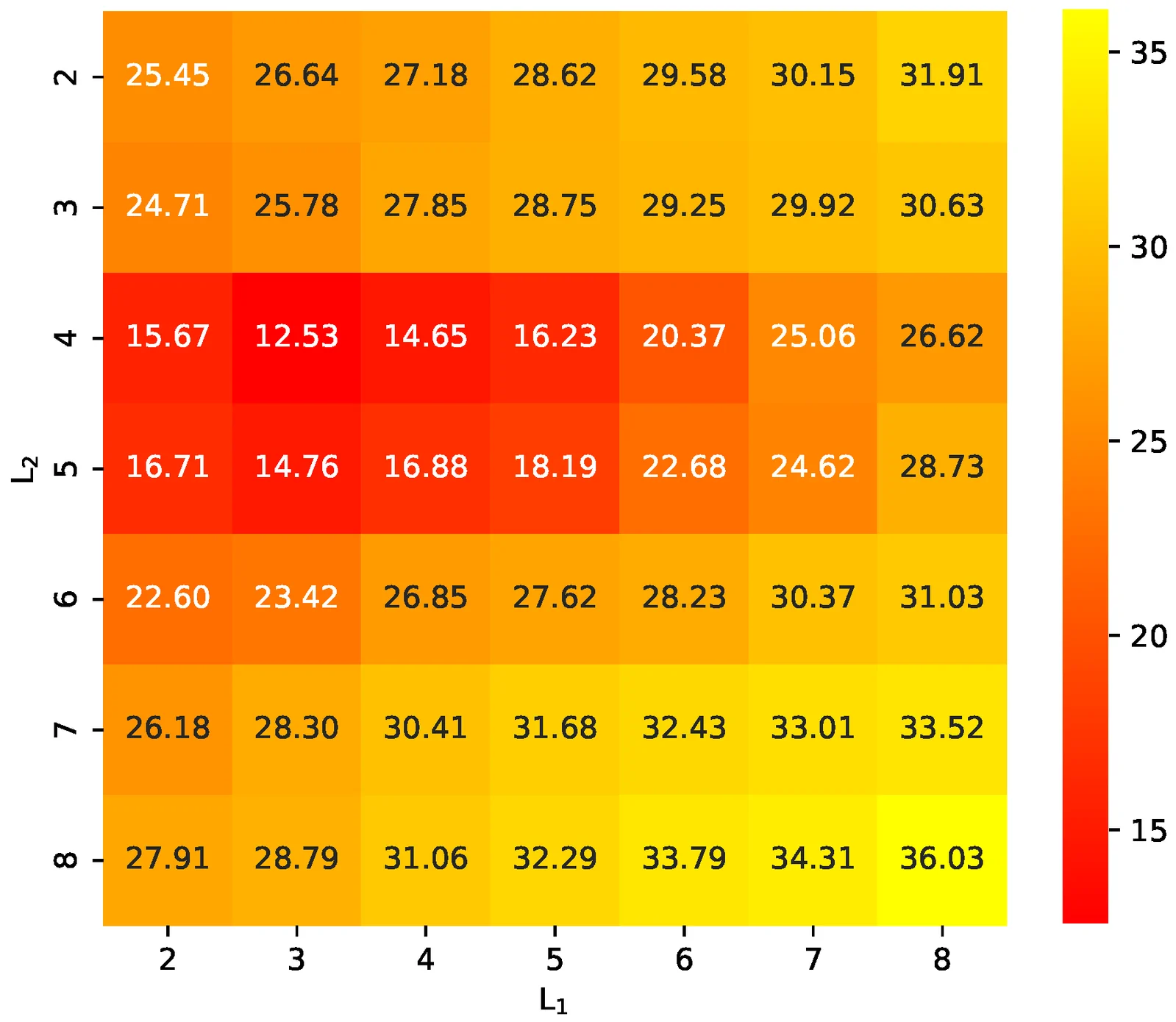
ACE (%) of Phase-PCANet with different number of filters.

Next, we examine the influence of filter quantity. Using a two-stage Phase-PCANet with fixed filter sizes of 5 × 5 for both stages, we systematically vary the number of filters in the first layer ($L_{1}$) and second layer ($L_{2}$) across a range of 2 to 8. The corresponding experimental results are shown in Figure 3.

From the results we can see that the ACE varies with the number of filters in both stages. The minimum ACE of 12.53% is achieved at ($L_{1}$,$L_{2}$) = (3,4), while the maximum ACE of 36.03% occurs at ($L_{1}$,$L_{2}$) = (8,8). In general, configurations with $L_{1}$ = 3 and $L_{2}$ = 4 yield the best performance, while larger filter numbers in both stages tend to increase the ACE. These results suggest that a moderate number of PCA filters is preferable for optimal fingerprint liveness detection.

**Figure 4:**
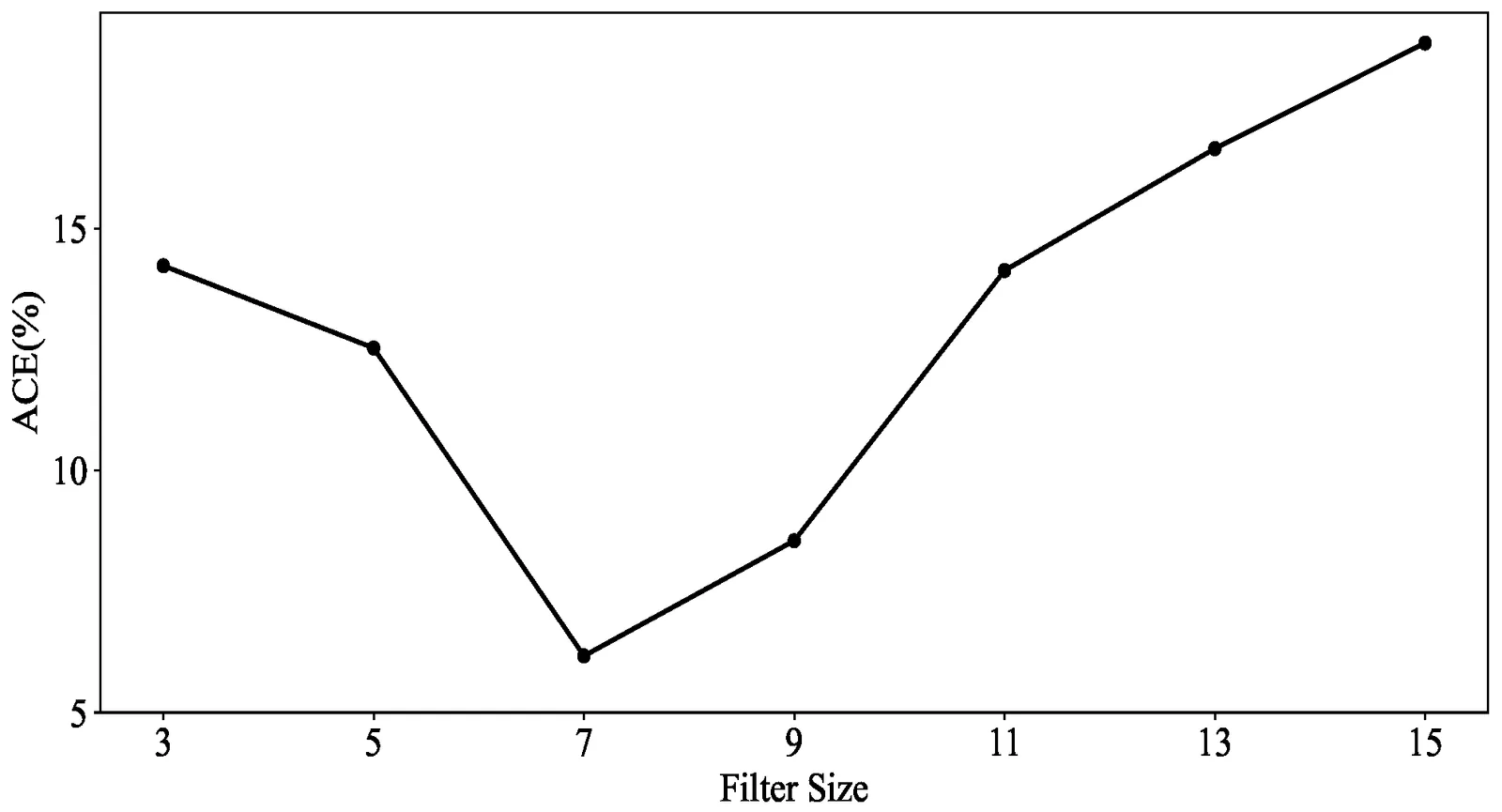
ACE of Phase-PCANet with different sizes of filters.

Finally, we examine the influence of filter size. Using the two-stage Phase-PCANet architecture with filter numbers fixed at ($L_{1}$,$L_{2}$) = (3,4), we vary the filter size [$k_{1}$; $k_{2}$] (with $k_{1}$ = $k_{2}$) from 3 to 15 in steps of 2. The results are shown in Figure 4.

The ACE of Phase-PCANet decreases as the filter size increases from 3 to 7, reaching its minimum at [7;7], and then rises steadily with further enlargement of the filter window. This trend indicates that a filter size that is too small fails to capture sufficient spatial context, while an excessively large size introduces redundant or noisy information that degrades discriminative performance.

### 4.4. Ablation Experiments

Ablation studies were performed to verify (1) the necessity of fusing local and global phase features, and (2) the effectiveness of the improved PCANet. Specifically, we performed ablation studies over six combinations of phase features (local, global, or fused) and network backbones (PCANet or improved PCANet), allowing us to quantify the distinct performance gains from feature fusion and the network enhancement. The experimental results are illustrated in Table 2.

The results in the table provide clear evidence for the efficacy of both proposed components. When using the original PCANet, fusing local and global phase features reduced the ACE from 10.37% (local only) and 11.42% (global only) to 9.92%, demonstrating that the two phase representations offer complementary information. Replacing the original PCANet with the improved version consistently lowered the ACE across all feature configurations, with reductions from 10.37% to 7.03% for local features and from 11.42% to 8.16% for global features. The full model, incorporating both feature fusion and the improved backbone, achieved the best ACE of 5.48%, substantially outperforming all other variants. These results confirm that both modifications contribute positively and synergistically to the final performance.

### 4.5. Intra-Sensor Evaluation

In this experiment, we evaluate the effectiveness of Phase-PCANet on three databases. All training and test data are from the same sensor. At the same time, the materials of forgery fingerprints in the test data also appeared in the training data. The experimental results are shown in Table 3, Table 4 and Table 5.

The proposed Phase-PCANet consistently outperforms all compared methods across the LivDet 2011, 2013, and 2015 databases. On LivDet 2011 (four sensors), Phase-PCANet achieves the lowest average ACE of 3.33%, substantially improving over the next best method, STRIVER (5.16%), and reducing the error by nearly 70% relative to the baseline LivDet 2011 (22.93%). On LivDet 2013 (three sensors), it attains an average ACE of 0.90%, surpassing STRIVER (1.40%) and all previous approaches, with particularly notable gains on the Swipe sensor (2.01% vs. 3.29%). On LivDet 2015 (four sensors), Phase-PCANet again ranks first with an average ACE of 5.48%, outperforming STRIVER (7.51%) and showing marked improvements on the GreenBit and CrossMatch sensors. Overall, Phase-PCANet demonstrates effective and robust generalization across different sensor types and acquisition conditions, achieving the lowest average classification error in all three intra-sensor benchmarks.

### 4.6. Cross-Material Evaluation

There are various materials involved in the production of forgery fingerprints, and the detection difficulties are also different. The cross-material liveness detection task is a practical problem that has to be solved in real-world scenarios. In this experiment, we use fingerprint images captured by the same sensor. Several spoof materials are held out from the training set as unseen materials, and are only evaluated during the testing phase. Experiments are carried out on LivDet2011 and compared with several methods with excellent performance. The results are shown in Table 6.

Experimental results on cross-material scenarios demonstrate that our proposed method achieves superior detection performance (lower ACE) over other state-of-the-art approaches, despite the presence of unseen fabrication materials in the test set.

### 4.7. Cross-Sensor Evaluation

In practice, fingerprint images requiring detection often originate from diverse sensors, exhibiting variations in resolution and image quality. Such heterogeneity poses significant challenges for liveness detection tasks. To rigorously assess the generalization capability of our proposed Phase-PCANet, we conduct cross-sensor experiments on unseen and unknown fingerprint samples. Specifically, the fingerprint images in the training and test sets were acquired from different sensors, thereby simulating real-world deployment scenarios. The experimental results are shown in Table 7.

The results show that all methods exhibit inevitable performance degradation due to sensor variability. Nevertheless, Phase-PCANet achieves the lowest error rates on four of the five evaluation settings and demonstrates clear advantages over the competing methods in the majority of cases. However, on the setting trained on Italdata2013 and tested on Biometrika2013, it underperforms relative to FLD-SRC and LCPD + WLD, indicating room for further sensor-invariant improvements.

### 4.8. Time Complexity Comparison

To comprehensively evaluate the efficiency of our proposed model, we conducted time complexity experiments on the LivDet 2015 database. Table 8 presents the average training time of different methods on this database.

The table compares average training times on the LivDet 2015 database. Traditional hand-crafted methods (e.g., LCP, WLBP, WLD) are significantly faster, ranging from 4.1 to 16.3 s. In contrast, deep learning models (CNN-VGG, DenseNet, AlexNet) require substantially more time, between 52.6 and 98.7 s. Our proposed Phase-PCANet achieves a training time of 20.1 s, which is notably lower than all deep networks and competitive even among hybrid approaches, striking a favorable balance between efficiency and representational power. We further measured the per-image inference time on a standard CPU platform (Intel i7-10700, 16 GB RAM). The average inference time per image on the LivDet 2015 database is 0.65 s.

A direct comparison with large-scale CNNs or ViTs is inequitable due to differences in data and hardware. Our Phase-PCANet, designed for data-efficient learning, achieves competitive results against deep baselines under limited data, confirming its value when large-scale pre-training is infeasible.

### 4.9. Discussion

#### 4.9.1. Phase-PCANet Performance Analysis

The quantitative results in Table 2, Table 3, Table 4, Table 5, Table 6, Table 7 and Table 8 establish the effectiveness of Phase-PCANet, yet several underlying factors merit further qualitative interpretation.

**Complementarity of the local and global phase.** The ablation study (Table 2) confirms that fusing the local and global phase consistently reduces ACE regardless of the backbone. The local phase (STFT-based) is sensitive to fine ridge textures and material-induced micro-anomalies, while the global phase (DFT-based) captures the holistic layout and periodicity of fingerprint patterns. These two representations are naturally complementary: the former excels at detecting spoof-related textural artifacts, whereas the latter provides robustness against local noise and structural distortion.

**Sensor-dependent performance.** Our method performs best on the Biometrika sensors (e.g., 0.26% ACE on LivDet 2013) and faces greater challenges on the Swipe sensor (2.01%), which produces narrow and motion-prone images. Despite this, Phase-PCANet achieves the best performance on almost all sensors and evaluation settings, demonstrating reasonable tolerance to resolution degradation and acquisition variability.

**Generalization to unseen materials and sensors.** Cross-material (Table 6) and cross-sensor (Table 7) experiments reveal inevitable performance drops, yet Phase-PCANet achieves the best or competitive performance against both handcrafted and CNN-based methods. We attribute this to the use of low-level phase statistics rather than material-specific high-level semantics, which reduces overfitting to known spoof materials and enhances sensor-invariant representation.

**Efficiency-accuracy trade-off.** With a training time of 20.1 s on LivDet 2015 (Table 8), Phase-PCANet sits between fast handcrafted descriptors (4.1–16.3 s) and heavy CNNs (52.6–98.7 s). The two-stage configuration ($F_{1,2}$) offers the best compromise, as additional stages yield either marginal improvements or clear performance degradation, while incurring substantially higher computational cost (Figure 2).

#### 4.9.2. Comparison with Phase Congruency

In addition to the phase representations adopted in this work, we note that phase congruency provides an alternative framework for structural feature detection that is robust to illumination and contrast variations [59,60]. Unlike raw Fourier/STFT phase, which is derived from a single-scale transform and is sensitive to window size and frequency selection, phase congruency measures the consistency of phase across multiple scales and orientations, typically implemented via log-Gabor filter banks. This yields feature maps that highlight perceptually meaningful edges, corners, and ridges with strong invariance to lighting changes.

In the context of fingerprint liveness detection, our empirical results indicate that raw local and global phase features—when combined with the improved PCANet—provide sufficient discriminative power to distinguish live from spoof fingerprints across multiple sensors and materials. The two-stage PCANet with dual binary coding effectively compensates for the lack of multi-scale phase aggregation by learning hierarchical filter responses from the phase maps.

However, we acknowledge that phase congruency offers potential advantages in cross-sensor and cross-material scenarios where illumination and contrast vary substantially. Its main drawback lies in computational cost: a full multi-scale phase congruency implementation typically requires convolution with filter banks across *S* scales and *O* orientations, resulting in S×O feature maps per pixel. In contrast, our local phase extraction involves only a single-window STFT followed by a closed-form 2×2 SVD per pixel, which is substantially lighter.

## 5. Conclusions

In this study, we propose Phase-PCANet, an image feature extraction method that jointly encodes local and global phase information within an improved PCANet framework for fingerprint liveness detection. Experimental evaluations on the LivDet 2011, 2013, and 2015 databases demonstrate the effectiveness of the proposed method.

However, cross-sensor performance remains unsatisfactory, revealing limited generalization under domain shifts. Future work will address this by incorporating phase congruency features and domain-adaptive feature alignment to mitigate distribution discrepancies. Beyond these improvements, inspired by recent advances in deep multi-level and multi-channel feature fusion [61,62], we intend to integrate phase-aware representations with more general architectures, including Transformer-based and CNN-based models, to improve scalability and cross-domain generalization.

## Figures and Tables

**Table 1:** Characteristic features of the LivDet 2011, 2013 and 2015 databases.

Database	Sensor	Image Size	Training	Testing	Spoofing Materials
			Real/Fake	Real/Fake	
LivDet 2011	Biometrika	372 × 208	1000/1000	1000/1000	Ecoflex, Gelatine, Latex, Siligum, Wood Glue
	Italdata	480 × 208	1000/1000	1000/1000	
	Digital Persona	391 × 208	1000/1000	1000/1000	Gelatine, Latex, Play-Doh, Silicone, Wood Glue
	Sagem	384 × 208	1000/1000	1000/1000	
LivDet 2013	Biometrika	352 × 384	1000/1000	1000/1000	Ecoflex, Gelatine, Latex, Modsil, Wood Glue
	Italdata	480 × 640	1000/1000	1000/1000	
	Swipe	1500 × 208	1221/979	1153/1000	
LivDet 2015	GreenBit	1000 × 1000	1000/1000	1000/1000	Ecoflex, Latex, Gelatine, Wood Glue, Liquid Ecoflex, RTV
	Biometrika	800 × 750	1510/1473	1500/1448	
	Digital Persona	500 × 500	1000/1000	1000/1500	
	Crossmatch	252 × 324	1000/1000	1000/1500	Body Double, Ecoflex, Play-Doh, OOMOO, Gelatine

**Table 2:** Ablation results on the LivDet 2015 database.

Feature	ACE (%)
Local Phase + PCANet	10.37
Global Phase + PCANet	11.42
Local-Global Phase + PCANet	9.92
Local Phase + improved PCANet	7.03
Global Phase + improved PCANet	8.16
Local-Global Phase + improved PCANet	5.48

**Table 3:** Performance comparison of different methods on the LivDet 2011 database.

Method	ACE (%)				
	BM	DP	ID	SA	Average
LivDet 2011 [45]	20.0	36.1	21.8	13.8	22.93
LCP [29]	-	-	-	-	33.21
WLBP [48]	6.6	4.4	11.1	3	6.275
WLD [49]	13.25	13.75	27.67	6.66	15.33
LPQ [18]	14.7	6.7	14.4	8	12.3
LBP [48]	13.0	10.8	24.1	11.5	14.85
MSLBP [19]	10.6	6.7	12.6	5.6	8.88
LCPD [25]	4.9	4.7	12.3	3.2	6.28
WLBD [48]	5.65	4.1	11.85	2.25	5.96
WCBF [50]	7.43	6.0	7.0	5.32	6.43
CEDD [51]	4.78	3.71	10.93	2.15	5.39
STRIVER [52]	4.46	3.50	10.61	2.06	5.16
Phase-PCANet	3.39	2.75	5.61	1.58	3.33

BM-Biometrika, DP-Digital Persona, ID-Italdata, SA-Sagem.

**Table 4:** Performance comparison of different methods on the LivDet 2013 database.

Method	ACE (%)			
	BM	ID	SW	Average
LivDet 2013 winner [53]	1.7	0.8	3.5	2
LCP [29]	-	-	-	27.95
WLBP [48]	1.05	2.2	7.2	3.48
WLD [49]	5.2	7.1	8.4	6.9
LBP [48]	1.6	3.0	10.1	4.9
HIG [20]	3.9	1.7	14.4	6.67
LCPD [25]	1.2	1.3	4.7	2.4
Dubey [26]	2.27	2.17	3.5	2.65
WLBD [48]	0.4	0.95	4.31	1.89
HyFiPAD [34]	-	-	-	2.85
IFPAD [35]	2.46	1.18	4.85	2.83
Densenet [54]	2.70	2.0	10.74	5.15
STRIVER [52]	0.32	0.58	3.29	1.40
Phase-PCANet	0.26	0.42	2.01	0.90

BM-Biometrika, ID-Italdata, SW-Swipe.

**Table 5:** Performance comparison of different methods on the LivDet 2015 database.

Method	ACE (%)				
	GB	BM	DG	CM	Average
titanz [47]	8.24	10.96	7.64	8.38	8.805
hbirkholz [47]	8.64	6.6	12	10.07	9.32
hectorn [47]	10	15.8	11.8	13.06	12.665
CSI MM [47]	13.44	24.44	12.16	10.01	15.01
CSI [47]	17.88	16.8	23.8	11.67	17.29
COPILHA [47]	27.24	20.04	24.36	31	25.66
DCM [13]	8.75	10.07	12.63	12.59	11.01
LBP [48]	7.88	13.48	14.44	11.46	11.82
WLD [49]	8.69	20.76	15.24	12.39	14.27
WLBD [48]	4.53	9.64	13.72	10.82	9.67
LCPD [55]	6.68	17.10	14.60	2.04	10.10
DRN [56]	13.58	13.37	16.68	8.87	13.12
STRIVER [52]	4.36	7.28	9.17	9.22	7.51
Phase-PCANet	3.29	6.17	9.03	3.43	5.48

GB-GreenBit, BM-Biometrika, DG-Digital, CM-CrossMatch.

**Table 6:** Cross-material performance comparison.

Sensor	Biometrika 2011	Biometrika 2013	ItalData 2011	ItalData 2013	Average
**Training**	EcoFlex, Gelatine, Latex	Modasil, Wood Glue	EcoFlex, Gelatine, Latex	Modasil, Wood Glue	–
**Testing**	Silgum, Wood Glue	EcoFlex, Gelatine, Latex	Silgum, Wood Glue, Other	EcoFlex, Gelatine, Latex	–
CNN-VGG [57]	10.10	4.90	22.10	6.30	10.85
LBP [57]	17.70	8.50	30.90	10.70	16.95
CNN-Alexnet [57]	12.2	5.8	25.80	8.0	12.95
LCPD+WLD [55]	10.65	4.35	11.70	3.85	7.63
FLD-SRC [58]	5.07	2.08	8.00	0.17	3.83
Phase-PCANet	4.42	1.73	7.29	0.15	3.39

**Table 7:** Cross-sensor performance comparison.

**Training**	Italdata2011	Italdata2011	Italdata2013	Biometrika2015	Italdata2011
**Testing**	Biometrika2011	Sagem2011	Biometrika2013	GreenBit2015	Italdata2013
CNN-VGG [57]	31	–	2.30	–	14.6
LCPD+WLD [55]	25.65	–	5.4	–	16.45
FLD-SRC [58]	24.85	29.27	16.5	30.31	12.25
Phase-PCANet	21.52	25.48	12.33	26.51	10.72

**Table 8:** Average training time (s) on the LivDet 2015 database.

Method	Average Training Time (s)
LCP	4.1
WLBP	5.3
WLD	6.0
MSLBP	5.6
LPQ	13.9
HyFiPAD	16.3
CNN-VGG	98.7
FLD-SRC	61.2
Densenet	52.6
CNN-Alexnet	56.7
Phase-PCANet	20.1

## Data Availability

The original contributions presented in this study are included in the article. Further inquiries can be directed to the corresponding author.
